# Oxidative Stress-Induced DNA Damage by Manganese Dioxide Nanoparticles in Human Neuronal Cells

**DOI:** 10.1155/2017/5478790

**Published:** 2017-05-17

**Authors:** Saud Alarifi, Daoud Ali, Saad Alkahtani

**Affiliations:** Department of Zoology, College of Science, King Saud University, Riyadh, Saudi Arabia

## Abstract

Metal nanoparticles have been extensively used in industry as well as in biomedical application. In this work, we have evaluated the toxic potential of manganese dioxide (MnO_2_) nanoparticles (MNPs) on human neuronal (SH-SY5Y) cells. Cellular toxicity due to MNPs (0, 10, 30, and 60 *μ*g/ml) on the SH-SY5Y cell was observed by 3-(4,5-dimethylthiazol-2-yl)-2,5-diphenyltetrazolium bromide (MTT) and neutral red uptake (NRU) tests. MNPs produced reactive oxygen species (ROS) and declined in mitochondrial membrane potential in the SH-SY5Y cell in dose and duration dependent manner. Moreover, lipid peroxide (LPO), superoxide dismutase (SOD), and catalase (CAT) activities were increased and glutathione was reduced in dose and time dependent manner. A significant upgrade in Hoechst 33342 fluorescence intensity (chromosome condensation) and phosphatidylserine translocation (apoptotic cell) was visualized in cells treated with MNPs for 48 h. On the other hand, caspase-3 activity was increased due to MNPs in SH-SY5Y cells. DNA strand breaks were determined by alkaline single cell gel electrophoresis assay (Comet Assay) and maximum fragmentation of DNA produced due to MNPs (60 *μ*g/ml) for 48 hours. This result provides a basic mechanism of induction of apoptosis and toxicity by MNPs in SH-SY5Y cells.

## 1. Introduction

Metal nanoparticles (NPs), with size of particle from 1 to 100 nm, possess unique physicochemical characteristics. Due to these characters, NPs are useful in the biomedical industry [1], as food additives [2] and cosmetics [3], for environmental applications [4], and in the construction industry [5]. The extensive application of metal NPs in various fields raises the risk of human exposure. Evaluation of the toxic effect of NPs to living organism is essential due to its hazardous effects on animal health [6]. Interactions of NPs to living cells induced diseases such as cancer and degenerative pathologies [7]. Discharge of high concentrated manganese is one of the main components of factories concerning production of steel and nonsteel alloy, colorants, battery manufacture, catalysts, and metal coatings. Olanow, 2004 [8], and Hussain et al. [9] reported that higher concentration of manganese is neurotoxic, causing neurological syndrome like Parkinson's disease.

Karmakar et al. [10] reported functional neurotoxicity in adult male Wistar rat after instilled manganese dioxide nanoparticles (MNPs) into the trachea for several weeks in daily doses of 2.63 mg/kg and 5.26 mg/kg. The main source of manganese exposure to human and animals is maneb contaminated fruits and vegetables and illicit drugs [11, 12]. Fell et al. [13] reported neuro- and hepatotoxicity in children due to manganese exposure. The neurotoxicity of MNPs (45-nm) in the brain and red blood cells, as determined through acetylcholinesterase activity, was significantly inhibited at doses of 1000 mg/kg and 500 mg/kg in female albino Wistar rats [14]. Hussain et al. [15] reported that cell membrane was damaged in BRL 3A rat liver cells at (100–250 *μ*g/ml) of MnO_2_. The human SH-SY5Y cell line was derived by subcloning from the parental metastatic bone tumor biopsy cell line SK-N-SH [16]. Despite the existing studies on the toxicity of MNPs, the underlying mechanism leading to toxicity remains unclear. A variety of nanoparticles including metal oxide particles induce reactive oxygen species (ROS) as one of the principal mechanisms of cytotoxicity [17]. ROS constitute a pool of oxidative species including superoxide anion, hydroxyl radical (OH^•^), hydrogen peroxide (H_2_O_2_), singlet oxygen (^1^O_2_), and hypochlorous acid. ROS are generated intrinsically or extrinsically within the cell. Molecular oxygen generates the primary ROS via one-electron reduction catalyzed by nicotinamide adenine dinucleotide phosphate oxidase.

End points of toxicity (oxidative stress, MTT, NRU, and ROS) which were chosen in the present study characterize vital metabolic functions of the mammalian system as well as provide general information of toxicity in a relatively acute exposure. The comet test is a sensitive technique for the measurement of DNA damage and alkali-labile sites in specific cells induced through different types of genotoxic agents [18].

So, in this study we have designed the experiment to observe genotoxicity and apoptosis in human SH-SY5Y cells due to MNPs.

## 2. Materials and Methods

### 2.1. Chemicals

Manganese oxide (MnO_2_) nanoparticles (MNPs 98.0%, APS ≤ 50 nm) were procured from US Research Nanomaterials, Inc. 3302 Twig Leaf Lane Houston, TX 77084, USA. 5,5-dithio-bis-(2-nitrobenzoic acid) (DTNB), MTT [3-(4,5-dimethylthiazol-2-yl)-2,5-diphenyltetrazolium bromide], neutral red (NR) dye, 2,7-dichlorofluorescin diacetate (H2-DCFH-DA), dimethylsulfoxide (DMSO), Annexin V FITC, Hoechst 33342, and propidium iodide were obtained from Sigma-Aldrich. Dulbecco's modified Eagle's medium (DMEM), fetal bovine serum (FBS), and antibiotics were purchased from Gibco, USA. All other chemicals used were of analytical grade and obtained from local suppliers.

### 2.2. Cell Culture

Human neuronal (SH-SY5Y) cells were obtained from ATCC, USA. SH-SY5Y cells were grown in Dulbecco's Modified Eagle Medium/F-12 nourished with fetal bovine serum (10%) and antibiotics (100 Unit/mL) in CO_2_ (5%) incubator at 37°C. After 80% confluence, SH-SY5Y cells were spilt into different size of flask and culture plates.

### 2.3. Characterization of MNPs

MNPs were dispersed in distilled water (1000 *μ*g/ml). Dispersed stock solution was mixed very well by using sonicator 40 W for 30 minutes. The size of MNPs was determined by field emission electron microscope (JEOL model 2100 F instruments) at an accelerating voltage of 200 kV. The particle size distribution of the MNPs was determined by dynamic light scattering (DLS, Model ZEN3600, Malvern Instruments Ltd., Malvern, UK).

### 2.4. Cell Morphology

SH-SY5Y cells (1 × 10^5^/well) were seeded in six-well plate and, after 24 h, cells were exposed to MNPs (0, 10, and 30 60 *μ*g/ml) for 24 and 48 h. Morphology of SH-SY5Y cells was observed after 24 and 48 h exposure by using an inverted microscope (Leica DMIL).

### 2.5. MTT Test

The MTT viability assay was performed with slight modifications as previously described by Mosmann [19]. SH-SY5Y cells were seeded in culture plates (96-well and 1 × 10^5^ cells per well) and incubated overnight at 37°C in 5% CO_2_ incubator. Cells were treated with MNPs (10, 30, and 60 *μ*g/ml) for 24 and 48 h and followed by the incubation MTT for 4 h. After removal of the medium, 100 *μ*l dimethyl sulfoxide (DMSO) was added to dissolve formazan crystals. The number of viable cells in each well was determined by quantifying the absorbance at 570 nm, with a reference wavelength of 630 nm, using a microplate spectrophotometer. All experiments were done three times with five replicates per experiment.

### 2.6. Neutral Red Uptake

NRU test has been done according to Borenfreund and Puerner [20] methods and measures the intake of the neutral red dye in lysosomes of viable cells. The cells (1.10^5^/well) were plated in 96-well plate and later 24 h; cells were exposed to different concentration of MNPs for 24 and 48 h. Neutral red dye (50 *μ*g) was dissolved in per ml medium and 0.2 ml of medium containing NR was added to each well. After 4-hour incubation of plate for intake of vital dye in viable cells the medium was discarded and cells were washed with fixative and dye extracting buffer from cells. After 15 minutes, microplate was agitated on shaker for 5 minute and absorbance of extracted dye was measured by microplate reader at 540 nm filter.

### 2.7. Mitochondrial Membrane Potential (MMP)

After exposure to MNPs (0, 10, 30, and 60 *μ*g/ml) for 24 h, MMP in SH-SY5Y cells was measured according to Rana et al. [21]. MMP were measured using the JC-1 cationic dye (Molecular Probes). SH-SY5Y cells were seeded in 96-well (black with the clear flat bottom) plate and cultured for one day. The cells were exposed to different concentration of MNPs for 24 h and incubated in Hank's balanced salts solution (HBSS) containing 2 *μ*g/ml JC-1 for 15 min. Green and red fluorescence was measured at 450 nm and 570 nm using a microplate reader, respectively. Depolarization of mitochondrial is assessed by the decline in ratio of red/green fluorescence intensity. For qualitative analysis of MMP, cell image was captured using fluorescence by upright microscope fluorescence (Nikon Eclipse 80i).

### 2.8. Measurement of ROS

Intracellular ROS generation in SH-SY5Y cells due to MNPs (0, 10, 30, and 60 *μ*g/ml) was measured by using H2-DCFH-DA dye as a detection reagent. In brief, H2-DCFH-DA (10 mM) in DMSO was diluted in DMEM/F as a working solution (5 mM). MNPs treated SH-SY5Y cells were incubated with H2-DCFH-DA (5 mM) in CO_2_ (5%) incubator at 37°C for 30 minutes; then cells were washed with normal PBS and fixed with paraformaldehyde (4%). Fluorescence of SH-SY5Y cells was observed by upright microscope fluorescence (Nikon Eclipse 80i).

SH-SY5Y cells (10^5^/well) were cultured in black bottom culture plate (96-well) and treated with MNPs over 48 h. After treatment, SH-SY5Y cells were incubated with H2-DCFH-DA (5 mM) for 30 minutes. Then they were washed with normal PBS. The intensity of fluorescence was quantified by using a dual-scanning microplate spectrofluorometer (Spectra MAX Gemini EM, Molecular Devices) at 480 nm excitation and 530 nm emission wavelengths. The results are presented as % of fluorescence intensity relative to control wells.

### 2.9. Oxidative Stress

SH-SY5Y cells (5 × 10^4^ cells/ml) were cultured in 75 cm^2^ culture flask for lipid peroxide (LPO), glutathione (GSH), superoxide dismutase (SOD), and catalase (CAT). After treatment with MNPs (0, 10, 30, and 60 *μ*g/ml) for 24 and 48 h, SH-SY5Y cells, scraped from flasks, were washed with PBS three times and agitated at 3000 rpm for 10 min at 4°C. Cells were resuspended in 0.3 ml of phosphate buffer saline and then sonicated on ice. The whole cell extract was centrifuged at 5000 rpm for 10 min at 4°C. Supernatant was used for the enzyme assays. The concentration of protein was quantified by Bradford method, using bovine serum albumin as standard [22], and presented in mg/ml.

Level of MDA, a biomarker for lipid peroxide, was tested through the thiobarbituric acid reaction [23]. A sample (200 *μ*l) with concentration of protein (2 mg/ml) was mixed with HCl (700 *μ*l, 0.1 N). After 20 min, it was mixed with thiobarbituric acid (TBA, 900 *μ*l of 0.025 M) and whole mixture was put in water bath for 60 min at 90°C. Finally, 0.1 M Tris-HCl (400 *μ*l), EDTA buffer (5 mM), pH 7.4 was added. The fluorescence of MDA was noted using a 520 nm/549 nm (excitation/emission) filter. A calibration curve with 1,1,3,3-tetramethoxypropane in the range 0.05–5 *μ*M was used to calculate the MDA concentration. The results were expressed as nmoles of MDA/mg protein.

The content of GSH in cell was measured by the colorimetric assay [24]. For intracellular GSH, supernatant was precipitated at 0.25% trichloroacetic acid and centrifuged at 12000 ×g for 10 min at 4°C. Twenty microliters from the protein precipitated sample was mixed with 160 *μ*l of 0.1 M phosphate-5 mM EDTA buffer, pH 8.3, and 20 *μ*l* o*-phthalaldehyde (OPT, 1 mg/mL in methanol) in a black 96-well plate. After 150 minutes of incubation at room temperature in the dark, fluorescence was measured at 460 nm (emission wavelength), in microplate spectrofluorometer (Spectra MAX Gemini EM, Molecular Devices) along with similarly prepared standards of GSH. Results are represented as GSH n mol/mg protein.

The activity of SOD was determined due to the oxidation of NADPH at 340 nm [7]. Hyperoxide anions were produced from molecular oxygen in the presence of mercaptoethanol EDTA and MnCl_2_. A control was done with each experiment of three triplicate samples. SOD activity (1 U) was supposed as the quantity of enzyme needed to inhibit the rate of NADPH oxidation of the control by 50%. SOD was represented in terms U/mg of protein.

Activity of CAT was measured by using spectrophotometer by observing the loss of H_2_O_2_ at 240 nm [25]. Its activity was determined in terms of U/mg protein, where 1 U was the quantity of enzyme that converted of 1 *μ* M H_2_O_2_ per minute.

### 2.10. Phosphatidylserine (PS) Translocation

Cells (5.10^4^) were grown in four-chamber precoated slides (Nunc). After exposure to MNPs, the cells were incubated in Annexin V-FITC (10 *μ*l) and propidium iodide (10 *μ*l) for 30 min. The cells were screened by a confocal microscope (CRCL's LSM 780 NLO confocal microscope) using Annexin V at 485 nm/518 nm (excitation/emission) and propidium iodide at 540 nm/617 nm (excitation/emission) under a 40x (NA = 1.4).

### 2.11. Chromosome Condensation

Chromosome condensation in SH-SY5Y cells due to MNPs (0, 10, 30 60 *μ*g/ml) treatment was detected by Hoechst 33342 staining. Hoechst 33342 solution was used to stain the exposed cells in six-well slides and the slides were incubated for 10 min in the dark at 37°C. Images of the nucleus were captured using a fluorescence microscope (Nikon).

### 2.12. Caspase-3 Assay

Caspase-3 was determined from the cleavage of N-acetyl-DEVD-p-nitroaniline (caspase-3 substrate I). The p-nitroaniline was considered as standard. Cleavage of the N-acetyl-DEVD-p-nitroaniline was observed at 405 nm, and finally the activity was represented in pmol nitroaniline/min/mg protein.

### 2.13. Alkaline Single Cell Gel Electrophoresis Assay (Comet Assay)

Comet test was done according Alarifi et al. [26]. Briefly, 70,000 cells/well were plated in a six-well plate. Cells were treated with MNPs (0, 10, 30 60 *μ*g/ml) over 48. After exposure cells were trypsinised and cells viability was observed [27] and samples showing cell viability more than 85% were proceeded for comet test. About 20 *μ*l of cell suspension (approximately 20,000 cells) was mixed with 80 *μ*l of low melting-point agarose (0.5%, LMPA) and made a layer on one end of a frosted plain glass slide, before it was layered with normal agarose (1%, 200 *μ*l, NA). Then, it was covered with a third layer of LMPA (100 *μ*l). After dryness of the gel, the slides were dipped in lysis buffer (2.5 M NaCl, 100 mM Na_2_ EDTA, 10 mM Tris pH 10 with 10% DMSO and 1% Triton X-100 added fresh) for 24 h at 4°C. Then slides were transferred in a horizontal gel electrophoresis unit, dipped in fresh cold alkaline electrophoresis buffer (300 mM NaOH, 1 Mm Na_2_ EDTA and 0.2% DMSO, pH > 13.5), and port in buffer for 20 min at 4°C for DNA unwinding and conversion of alkali-labile sites to single strand breaks. Electrophoresis was done in same buffer at 4°C for 20 min, by using 15 V (0.8 V/cm) and 300 mA. The slides were washed with 0.4 M Tris buffer at (pH 7.5) to remove excess alkali and then stained with EtBr (20 *μ*g/ml, 75 *μ*l) for 5 min for observation of DNA strand breakage. The cells were exposed ex vivo with 100 *μ*M H_2_O_2_ for ten min at 4°C for positive control. The slides (2) per concentration were prepared and 100 cells per slide (200 cells/concentration) and the slides were randomly scored using an image analysis system (Komet-5.5, Kinetic Imaging) attached to a fluorescent microscope (Leica) equipped with appropriate filters. The parameter selected for quantification of DNA damage was percent tail DNA (i.e., % tail DNA = 100 − % head DNA) and olive tail moment as determined by the software.

### 2.14. Analysis of Data

Three independent experiments were done. Each dose was tested in duplicate, in each experiment. Results are presented as average ± standard errors. Analysis of statistic was done by using analysis of variance (ANOVA) by using SPSS software. The difference was taken significant (*p* < 0.05).

## 3. Results

### 3.1. MNPs

**Figure 1:**
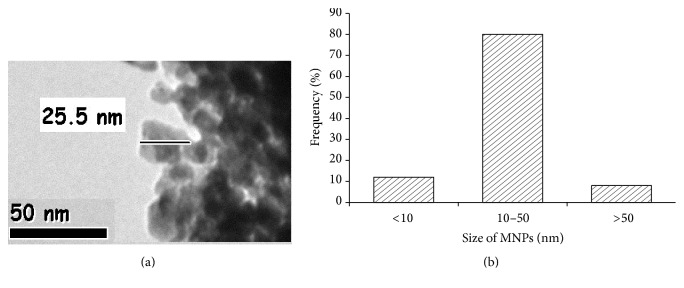
Transmission electron microscopy (TEM) images of manganese oxide nanoparticles (MNPs) (a) and size distribution diagram of MNPs (b).

The mean size of MNPs is around 40.6 ± 2.4 nm and it shows maximum NPs were in round shape (Figure 1). Mean hydrodynamic size and zeta potential of MNPs in water were measured by DLS and it was 299.60 nm and −7.2 mV, respectively.

### 3.2. Alteration in Morphology of SH-SY5Y Cells and Cell Viability

**Figure 2:**
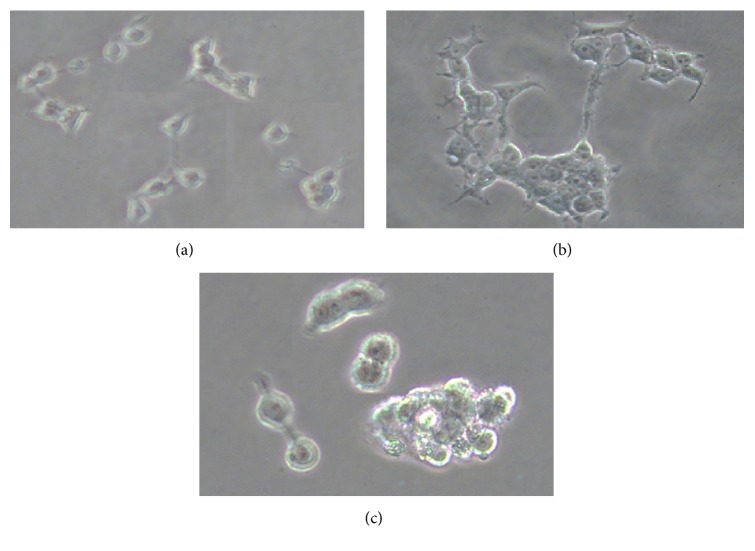
Morphology of SH-SY5Y cells. (a) Control cell, (b) exposed cells at 30 *μ*g/ml of MNPs, and (c) exposed cells at 60 *μ*g/ml of MNPs 48 h. Number of experiments (*n* = 3).

Figure 2 showed the comparative morphology of unexposed and MNPs treated SH-SY5Y cells for 24 and 48 h. SH-SY5Y cells treated MNPs (60 *μ*g/ml) changed into round shape and detached from the surface (Figure 2(b)).

**Figure 3:**
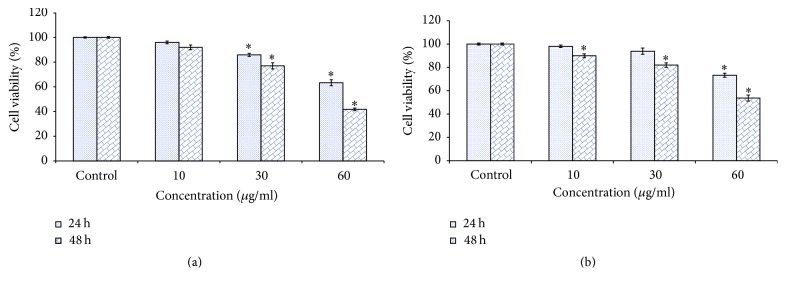
Cytotoxicity of MNPs in SH-SY5Y cells for 24 and 48 h, as determined by (a) MTT and (b) NRU tests. Each value represents the mean ± SE of three experiments. *n* = 3, ^*∗*^*p* < 0.05 versus control.

MNPs induced cell toxicity in SH-SY5Y cells was observed as a mitochondrial function (MTT reduction) and lysosomal damage (NR uptake). MTT results showed cytotoxicity in a dose and time dependent manner (Figure 3(a)). NR uptake, a marker of lysosome damage, was estimated in SH-SY5Y cells after exposure MNPs for 48 h. However, as the dose and time of MNPs exposure increased, the significant NR uptake was recorded (Figure 3(b)).

### 3.3. ROS Generation and Oxidative Stress

**Figure 4:**
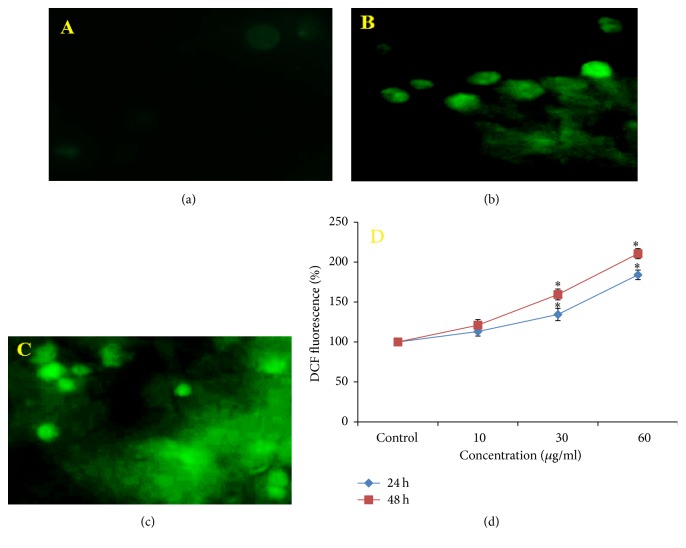
MNPs induced ROS in SH-SY5Y cells. (a) Control, (b) at 60 *μ*g/ml of MNPs for 24 h, (c) at 60 *μ*g/ml of MNPs for 48 h, and (d) % ROS production in SH-SY5Y cells. Each value represents the mean ± SE of three experiments. *n* = 3, ^*∗*^*p* < 0.05 versus control.

Induction of oxidative stress by MNPs was evaluated by estimation of the ROS, LPO, GSH, SOD, and CAT levels in SH-SY5Y cells. Results showed that MNPs induced a significant (*p* < 0.05) qualitative and quantitative dose and time dependent increase in ROS generation (%) was observed in the form of fluorescence on treatment with MNPs (13%, 34.6%, and 84.3% for 24 h and 20.86%, 59.45%, and 110.6% for 48 h at 10, 30, and 60 *μ*g/ml respectively; Figures 4(a), 4(b), 4(c), and 4(d)).

**Figure 5:**
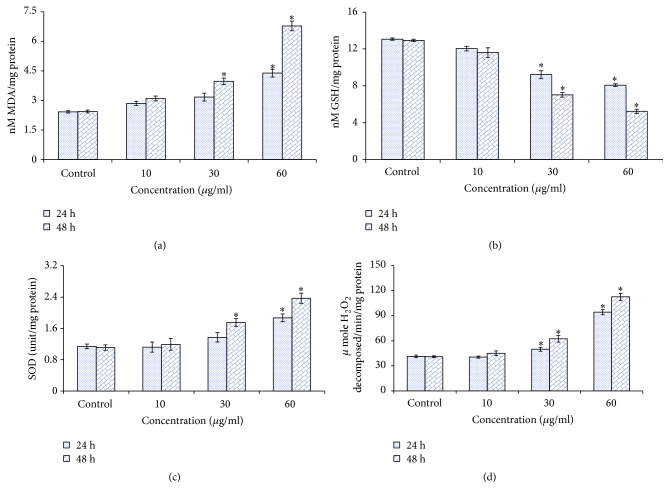
(a) Levels of LPO, (b) GSH, (c) SOD, and (d) CAT in SH-SY5Y cells after exposure to MNPs for 24 and 48 h. Each value represents the mean ± SE of three experiments. *n* = 3, ^*∗*^*p* < 0.05 versus control.

Cells treated with MNPs showed a dose and time dependent statistically significant (*p* < 0.05) increase in LPO at dose 30 *μ*g/ml (15.3% for 48 h) and at 60 *μ*g/ml (19.7% for 24 h and 43.40% for 48 h Figure 5(a)).

A significant reduction (*p* < 0.05) in cellular GSH content was observed in dose and time dependent manner after 24 and 48 h exposure to MNPs in human neuronal cells (Figure 5(b)).

An elevation of SOD and CAT activities was found in dose and time dependent manner after MNPs exposure (Figures 5(c) and 5(d)).

### 3.4. MNPs Induced Mitochondrial Membrane Potential (MMP)

**Figure 6:**
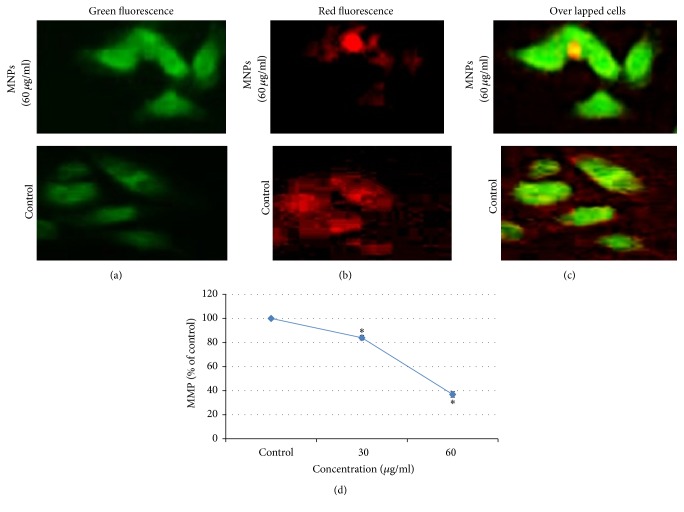
Induction of MMP in SH-SY5Y cells at 60 *μ*g/ml of MNPs exposure. (a) Indicates green fluorescence (JC-1 monomer); (b) Indicates red fluorescence (JC-1 aggregate); (c) Indicates overlapped cells (green-red fluorescence); (d) % MMP ratio. Data are expressed as the mean ± SE of three experiments. *n* = 3, ^*∗*^*p* < 0.05 versus control.

We have evaluated effect of MNPs on the MMP of SH-SY5Y cells using the dye, JC-1. After exposure of MNPs caused a complete collapse of mitochondrial membrane potential as indicated by the decrease in aggregates (red) and increase in monomers (green) (Figures 6(a), 6(b), 6(c)), MNPs decreased mitochondrial membrane potential in cells as measured by a fluorescent plate reader (Figure 6(d)).

### 3.5. Chromosome Fragmentation, Caspase-3 Activity, and Phosphatidylserine Translocation

**Figure 7:**
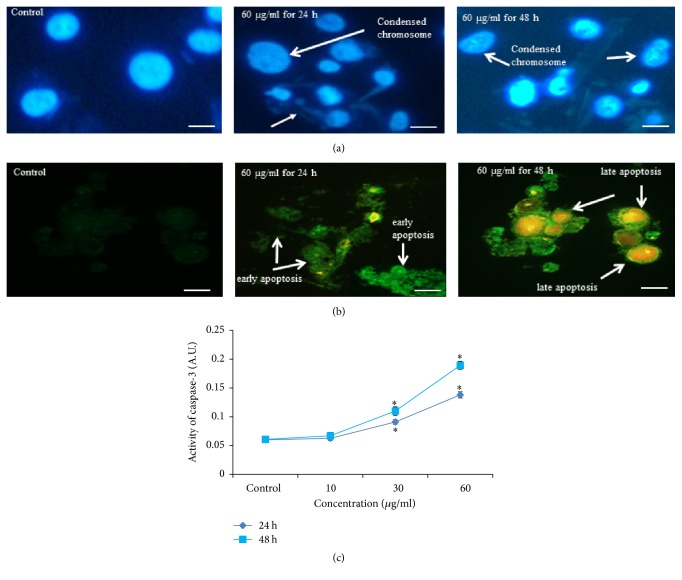
(a) Chromosomal condensation at 60 *μ*g/ml for 24 and 48 h; (b) MNPs induced apoptosis as demonstrated by phosphatidylserine translocation in SH-SY5Y cells incubated with different concentrations of MNPs exposure for 24 and 48 h and stained with Annexin V and propidium iodide (PI); (c) induction of caspase-3 activity in SH-SY5Y cells after exposure to MNPs for 24 and 48 h. Each value represents the mean ± SE of three experiments. *n* = 3, ^*∗*^*p* < 0.05 versus control. Scale bar 10 *μ*m.

After exposure to MNPs, fragmentation of chromosome (Figure 7(a)) and phosphatidylserine translocation (Figure 7(b)) was observed in SH-SY5Y cells.

When cells were treated with MNPs for 24 and 48 h, the activity of caspase-3 was increased in dose and time dependent manner (Figure 7(c)).

### 3.6. DNA Damage

**Figure 8:**
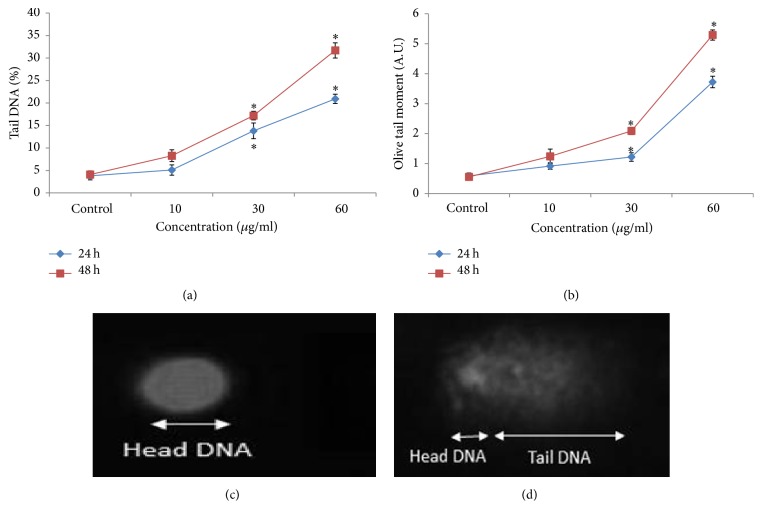
DNA fragments in SH-SY5Y cells due to MNPs. (a) Tail DNA (%), (b) olive tail moment, (c) control cell, and (d) exposed cell to MNPs (60 *μ*g/ml) for 48 h. Each value represents the mean ± SE of three experiments. *n* = 3, ^*∗*^*p* < 0.05 versus control.

The DNA damage was measured as % tail DNA and olive tail moment in the control and MNPs treated SH-SY5Y cells. SH-SY5Y cells exposed to MNPs exhibited significantly higher DNA damage in cells than control. The highest DNA damage was recorded at 60 *μ*g/ml MNPs in SH-SY5Y cells (Figure 8).

## 4. Discussion

There are different types of metals, for example, iron, manganese, mercury, copper, lead, and zinc, widely used in industry and animal or human is exposed at the workplace or as a consequence of contaminated food and environment. Ferrer [28] reported that short or long-term exposure to metals produced mutagenic disease. Manganese exposure increased the threat of nervous system disorder and Parkinson's disease [29]. The aim of this study was to find out the toxic effect of MNPs in SH-SY5Y cells. Morphological changes were observed as a loss of neurites and cell adhesion, rounding and swelling of cell bodies, and finally rupture and lysis of the cytoplasmic membrane in accordance with increasing MNPs concentration and time of exposure; considerable damage was evident upon application of ≥30 *μ*g/ml. We have measured cell toxicity of MNPs by two tests such as NRU and MTT tests to validate the result. ROS generation ability of NPs belongs to the cell toxicity in different types of cells [30]. Generation of ROS has been observed in different metal and nonmetal NPs as carbon nanotubes and fullerenes. ROS and free radical generation are one of the specific reasons of toxicity of NPs; as a consequence oxidative stress and inflammation occur and cause damage to cell organisms [31]. In present result, we have found increasement of ROS in MNPs treated SH-SY5Y cells through H_2_DCFDA. The production of ROS and oxidative stress as observed in the present study is in accordance with the findings of Wilson et al., [32] in the primary astrocytes for NPs. MNPs reduced the viability of SH-SY5Y cells, enhanced production of ROS, MDA, SOD, and CAT levels, and decreased GSH levels. NPs have been recognized as an activator of autophagy cell death [33]. Enhancement of ROS generation may distract health of organism due to oxidative injury activating senescence. A collection of oxidative damage as a consequence induces mitochondrial damage [34]. We have observed damage of mitochondrial membrane potential after MNPs exposure in human neuronal cells. It may occur due to NPs-induced oxidative stress in cells. Some researchers reported that oxidative stress and DNA damage were involved in the toxic effects of silica NPs on human neuronal cells (SH-SY5Y) [35].

We have measured apoptotic enzyme activity as caspase-3 in SH-SY5Y cells after exposure of MNPs. Caspase-3 activity was increased as the doses of MNPs increase. We assessed a rise in LPO, SOD, and CAT and a drop in GSH level in SH-SY5Y cells after treating with MNPs, which shows a marker of oxidative stress. LPO can give rise to more radicals and fragments biomolecules with ROS. MNPs induce injury to the cell lysosome as demonstrated by NRU uptake. Decline in GSH in MNPs treated SH-SY5Y cells combined with the augmented level of SOD, CAT, and LPO presents that oxidative stress can be the initial background of MNPs in SH-SY5Y cells. When free radicals come in close with the cellular organelles, they may oxidize and reduce macromolecules (DNA, lipids, and proteins) resulting in significant oxidative damage to the cell. MNPs reduced cell viability, enhanced ROS production, decreased the glutathione level, inhibited LPO level, and increased apoptosis in SH-SY5Y cells. DNA damage, determined by the comet assay, was detected in the SH-SY5Y cells after cells were exposed to MNPs. The DNA damage and oxidative stress as observed in the present study are in accordance with the findings of Alarifi et al. [26] in the human hepatocarcinoma cells for nanoalumina and Alkahtane [36] in the A549 for indium tin oxide nanoparticles.

In conclusion, the data confirm that MNPs have the capacity to induce apoptosis and DNA fragmentation in human neuronal cells. Our acute exposure study of high-level stimulation of apoptotic and oxidative stress of MNPs will need to be further investigated to determine whether long-term exposure consequences may exist for MNPs application.
